# Polymeric Carbon Nitride Armored Centimeter-Wide Organic Droplets in Water for All-Liquid Heterophase Emission Technology

**DOI:** 10.3390/polym12081626

**Published:** 2020-07-22

**Authors:** Qian Cao, Baris Kumru

**Affiliations:** Department of Colloid Chemistry, Max Planck Institute of Colloids and Interfaces, 14424 Potsdam, Germany; qian.cao@mpikg.mpg.de

**Keywords:** carbon nitride, waterborne systems, multicolor emission droplets, carbon nitride interfaces, all liquid displays

## Abstract

High potential of emission chemistry has been visualized in many fields, from sensors and imaging to displays. In general, conjugated polymers are the top rankers for such chemistry, despite the fact that they bring solubility problems, high expenses, toxicity and demanding synthesis. Metal-free polymeric semiconductor graphitic carbon nitride (g-CN) has been an attractive candidate for visible light-induced photocatalysis, and its emission properties have been optimized and explored recently. Herein, we present modified g-CN nanoparticles as organodispersible conjugated polymer materials to be utilized in a heterophase emission systems. The injection of a g-CN organic dispersion in aqueous polymer solution not only provides retention of the shape by Pickering stabilization of g-CN, but high intensity emission is also obtained. The heterophase all-liquid emission display can be further modified by the addition of simple conjugated organic molecules to the initial g-CN dispersion, which provides a platform for multicolor emission. We believe that such shape-tailored and stabilized liquid–liquid multicolor emission systems are intriguing for sensing, displays and photonics.

## 1. Introduction

Light emission with spatial control is the base of imaging, optoelectronics and active displays [1]. Typically, advanced π-conjugated polymers that are capable of light absorption and emission are used, e.g., in OLEDS or for optical superresolation microscopy [2,3,4]. Such experiments can be conducted from a dissolved single phase or from a colloidal state using particulate dispersions [5,6,7,8]. However, solubility and processing of these polymers relies on conjugation with solution-promoting entities, such as side chain modification. Polymeric graphitic carbon nitride (g-CN) is a potential replacement and has striking advantages, such as low cost, non-toxicity and ease of tenability [9]. g-CN is ideally composed of carbon and nitrogen in tri-s-triazine repeating units to form a conjugated polymer structure, and can be synthesized from simple nitrogen-rich commodity molecules such as melamine and urea by thermal condensation at around 500 °C [10]. In addition, the supramolecular complexation of monomers renders a class of carbon nitride materials with special morphologies and photophysical properties [11,12,13]. For example, carbon nitride nanosheets can be attained by the thermal condensation of acid-treated melamine cyanurate complex at 650 °C [14]. g-CN ideally has surface defects and a negative surface charge, and can easily be modified via pre- or post-modification methods [15]. Visible light-induced photoactivity of g-CN is instrumentalized for water splitting [16,17,18,19,20], CO_2_ reduction [21,22,23,24], polymer synthesis [25,26,27], and solar cells [28,29,30]. Emission color and intensity of g-CN materials can be tailored via monomer engineering, which provides an interesting platform to be investigated. For example, phenyl edges onto g-CN can be introduced, and synthesis at lower temperature (450 °C) results in incomplete condensation (nanosheets), and like that, water dispersible and highly fluorescent g-CN can be attained (labeled as CMp) [31]. Furthermore, the emission color of CMp dispersion can be adjusted to multicolor emission by the introduction of water-soluble π-conjugated polymers with different side chains [32]. g-CN however shares the same weakness as other π-conjugated analogues, i.e., high polarization and the coupled problems for solubility. Strong stacking in general prevents the preparation of dispersions, especially in organic media. A recent development by our group solved this problem and presented highly stable organodispersions of g-CN, using light-induced vinyl thiazole modification (denoted as CMp-vTA). In these systems, a permanent charge separation is created, and electrostatic forces repel the single conjugated sheets [33].

Surface properties of g-CN are highly intriguing. g-CN is not only a light active polymer, but was also shown to be a Pickering stabilizer for oil-in-water emulsions [34]. This indicated the possibility of conducting chemistry at the water-oil interfaces using g-CN both as stabilizer and catalyst, for hydrogenation reactions or light-induced emulsion polymerization [35,36,37]. Fruitful combination of g-CN surface chemistry linked to interfaces might pave the way for novel applications beyond photocatalysis.

Aqueous soft materials have been of great interest, and the persistent materials could be manufactured via crosslinking and host-guest complexation [38,39,40,41]. A switch from single phase materials to heterophase systems in order to generate interface-induced soft materials has been a trend recently. This methodology is based on utilizing oppositely charged molecules dissolved in the different phases, and during the contact, interfaces are formed, which are immediately stabilized by interfacial polyelectrolyte complex formation. As a result, soft and elastic films are formed at the interface, which provide a retention of the shape of the injected phase, while the inner part is still liquid [42]. Since the first description, different combinations of molecules were proposed by Russell and colleagues, and systems mainly rely on injecting aqueous phases in organic phases [43,44,45]. Yet, this approach provides stimulation to envision novel soft, structured, liquid devices and novel display technologies.

In this report, we will exhibit the combination of emissive properties of g-CN harnessed with its surface properties to form soft emissive droplets. We will demonstrate the formation of organic g-CN dispersion droplets in water via simple syringe injection. Furthermore, we will present the tunability of g-CN emission by introducing simple aromatic molecules to the initial g-CN dispersion, and how the system can be manipulated towards all-liquid heterophase multicolor emission.

## 2. Materials and Methods

### 2.1. Chemicals

2,4-diamino-6-phenyl-1,3,5-triazine (97%, Sigma Aldrich, Darmstadt, Germany), 4-methyl-5-vinylthiazole (vTA, 97%, Sigma Aldrich), anthracene (>96%, Sigma Aldrich, Germany), chloroform (99%, Merck, Germany), cyanuric acid (98%, Sigma Aldrich), ferrocene (98%, Sigma Aldrich, Germany), poly(ethylene imine) (PEI, *M_n_* 60,000 g/mol, 50 *wt%* in water, Sigma Aldrich), pyrene (98%, Sigma Aldrich, Germany).

### 2.2. Synthesis of CMp-vTA

CMp-vTA was synthesized according to the literature [33] from cyanuric acid (C) and 2,4-diamino-6-phenyl-1,3,5-triazine (Mp) supramolecular complex followed by 4-methyl-5-vinylthiazole (vTA) photomodification. 

### 2.3. Droplet Formation Experiments

Prior to the experiment, CMp-vTA was dispersed in chloroform (40 mg ml⁻^1^) via sonication for 30 min. Afterwards, dispersion was collected into a syringe and injected in a water bath with PEI (80 mg ml⁻^1^) at room temperature to form stable centimeter-sized droplets.

For color tuning of droplets, certain amounts of pyrene, anthracene and ferrocene (14 mg ml⁻^1^) were added to CMp-vTA dispersion, and droplet formation was achieved as explained previously.

### 2.4. Characterization

Scanning electron microscopy (SEM) was performed on a Jeol JSM 7500 F (Tokyo, Japan) equipped with an Oxford Instruments X-MAX 80 mm^2^ detector (Abingdon-on-Thames, UK). Solid state ultraviolet-visible (UV-Vis) spectroscopy was recorded via a Aglient Cary 500 Scan spectrophotometer equipped with an integrating sphere (Waldbronn, Germany). Photoluminescent emission spectra were recorded on a Jasco FP-8300 (Pfungstadt, Germany) instrument at ambient temperature with the excitation wavelength at 360 nm. Transmission electron microscopy (TEM) measurements were acquired using a double-corrected Jeol ARM200F (Tokyo, Japan), equipped with a cold field emission gun and a Gatan GIF Quantum. The used acceleration voltage was 200 kV and the emission was set to 10 μA in order to reduce beam damage. Sample was dispersed in dichloromethane and evaporated on active surface prior to TEM measurement. Combustive elemental analysis of CMp-vTA was recorded via a Vario Micro device (Langenselbold, Germany). Florescence of the droplets was excited by Vilber Lourmat brand Ultra-violet radiation (15 W) source with excitation wavelength of 365 nm (Marne-la-Vallee, France).

## 3. Results

### 3.1. Synthesis and Characterization of CMp-vTA

**Figure 1:**
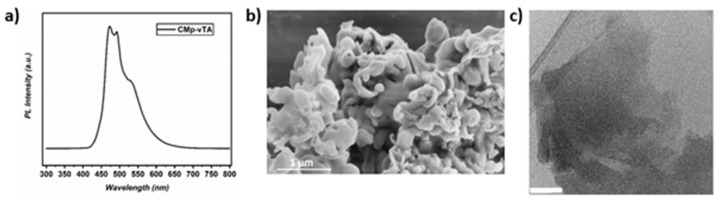
(**a**) Photoluminescence (PL) spectra of CMp-vTA; (**b**) SEM image of CMp-vTA; (**c**) transmission electron microscopy (TEM) image of CMp-vTA nanosheets (scale bar corresponds to 50 nm).

Organodispersible CMp-vTA was synthesized via one pot photoinduced surface grafting on CMp as reported previously [33]. Photoluminescence (PL) spectra of CMp-vTA proves the formation of excited state and corresponding photoluminscence under UV illumination (Figure 1a). A SEM image of CMp-vTA shows rather uniform morphology without any special features (Figure 1b) and TEM reveals that CMp-vTA consists of sheets that are 100–150 nm in size (Figure 1c). Combustive elemental analysis resulted in 49.3% N, 44.6% C and 1% S, indicating that the thiazole species are present in the structure (from the sulfur content). We have attempted to measure the size distribution of particles by dynamic light scattering, however TEM provides better insight to estimate the sheet size of carbon nitride materials.

### 3.2. Formation of Organic Droplets in Aqueous Phase via Injection

In the next step, a chloroform dispersion of CMp-vTA was prepared, and injection into water was performed. The aqueous phase contains PEI to provide potential polyelectrolyte complex formation at the interface, as long PEI chains are expected to interlink the CMp-vTA at the interface from the water phase, thus resulting in jamming of the sheets. It is important to underline that attempts to utilize pure water have not allowed the creation of persistent droplet shapes, therefore the cationic polymer in water is needed. Chloroform was chosen as it has a higher density than water, so the created particles or structures do not float. The chloroform dispersion of CMp-vTA was prepared via sonication in a low intensity sonic bath for 30 min, and was subsequently injected into the aqueous phase.

**Figure 2:**
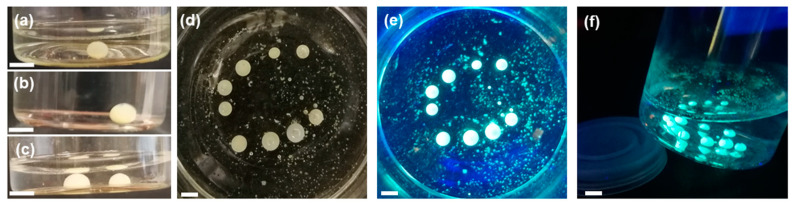
Digital images of CMp-vTA/chloroform (CN/TCM) droplet in aqueous PEI solution at (**a**) pH = 2; (**b**) pH = 7; (**c**) pH = 9; (**d**) macroscopic ‘c’-shaped organic droplets; (**e**) fluorescent image of ‘c’-shaped organic droplets (**f**) fluorescent image of tilted glass vial containing ‘c’-shaped organic droplets. Scale bars correspond to 1 cm.

We chose a syringe to form droplets as this in principle was similar to inkjet or melt printing. Injection at different pH values was conducted; droplets are persistent from acidic conditions (pH = 2, Figure 2a) over neutral pH (Figure 2b) to slightly basic conditions (pH = 9, Figure 2c), while higher basicity leads to destruction of the droplet shape. Therefore, one can conclude that the formation and stability of the interface layer depends on complex formation between differently charged colloids or polymers, which is similar to previous reports from the literature [46]. Under the applied conditions, PEI was protonated and underwent interfacial interactions with the negatively charged CMp-vTA contained in the organic droplet. It is possible to form stable droplets by injection, and a pattern such as a ‘c’ was formed to demonstrate the robustness of the created structures (at neutral pH with PEI, Figure 2d). Furthermore, high intensity light emission by CMp-vTA is activated by UV illumination (Figure 2e). The stability of the droplets against minor mechanical disturbances is an important issue. To illustrate the moderate yield stress, we tilted the glass vial that contained droplets (Figure 2f), and the droplets stayed bound to the bottom. Therefore, stable emissive soft droplets can be harnessed by simple interface complexation chemistry.

**Figure 3:**
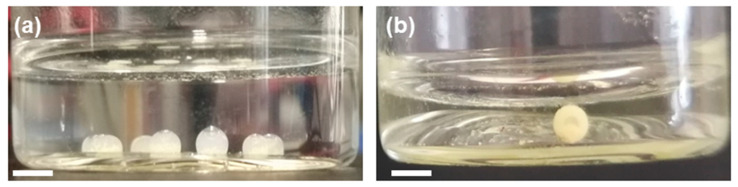
(**a**) Digital image of CMp-vTA/chloroform (CN/TCM) ‘c’-shaped droplets in aqueous PEI solution overnight (side view); (**b**) digital image of CMp-vTA/chloroform (CN/TCM) droplet in aqueous PEI solution at pH = 2 after 3 days (side view). Scale bars correspond to 1 cm.

The stability test was performed by leaving ‘c’-shaped organic droplets undisturbed overnight, and the droplets remained unchanged (Figure 3a). Furthermore, a stability test was conducted at pH = 2 for a single droplet over 3 days (Figure 3b), and the stability and viability of the presented methodology based on organic CMp-vTA dispersion was confirmed. 

### 3.3. Multicolor Emission

**Figure 4:**
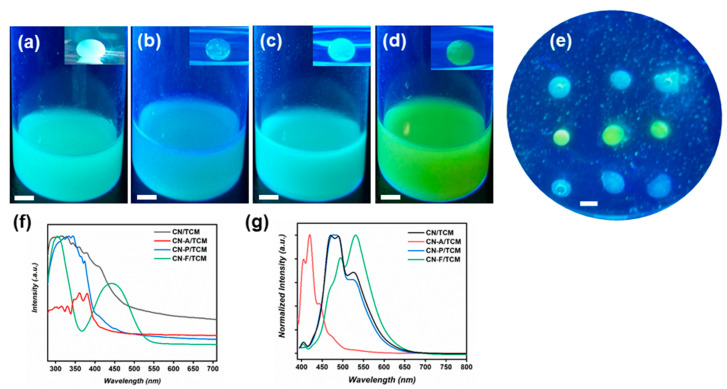
Series of digital images under UV (**a**) CMp-vTA/chloroform (CN/TCM) dispersion and corresponding droplet (inlet); (**b**) CMp-vTA+anthracene/chloroform (CN-A/TCM) dispersion and corresponding droplet (inlet); (**c**) CMp-vTA+pyrene/chloroform (CN-P/TCM) dispersion and corresponding droplet (inlet); (**d**) CMp-vTA+ferrocene/chloroform (CN-P/TCM) dispersion and corresponding droplet (inlet); (**e**) droplets from inlets of b (bottom), c (top) and d (middle) together. Scale bars correspond to 1 cm. (**f**) UV-Vis spectra of utilized dispersions and (**g**) emission spectra of utilized dispersions.

Accessing multicolor emission by simple adjustments of one target system is highly desirable. As it has been previously demonstrated for perovskite systems, changing the halide ion of the perovskite structure reveals altered emission colors [7]. Likewise, non fully-condensed carbon nanostructures possess multiple color emission properties based on the synthesis conditions [47]. g-CN materials possess emission, but the inherent emission color is mainly restricted to blue and green [48,49]. Charge transfer to a second fluorophore is the second possibility to alter the emission color. For g-CN materials, such an approach was previously described in aqueous systems with high-specialty conjugated polymers with diverse backbones, but we strived for something simpler and cheaper [32]. Hybridization of CMp-vTA with small aromatic molecules was found to alter emission properties in a similar fashion, therefore anthracene, pyrene and ferrocene were chosen as shifting agents. Addition of those molecules (14 mg ml⁻^1^) to CMp-vTA dispersion alters the emission properties visibly (Figure 4a–d). Correspondingly, injecting such dispersions into aqueous PEI solution provides multicolored emissions in a facile fashion (Figure 4a–d inlets).

In order to quickly visualize the potential, we have employed droplets from two different dispersions. When droplets were utilized together, individual colors were still detectable (Figure 4e). This was further examined by UV and PL measurements of employed dispersions (Figure 4f,g). Absorbance of the dispersions differed from each other, and shifts could be obtained in emission spectra, except for pyrene. Similar emission spectra after pyrene integration might indicate a more complex charge transfer-accumulation mechanism between CMp-vTA and pyrene, similar to carbon nitride-perylene [50], however it is outside of the scope of this project.

**Figure 5:**
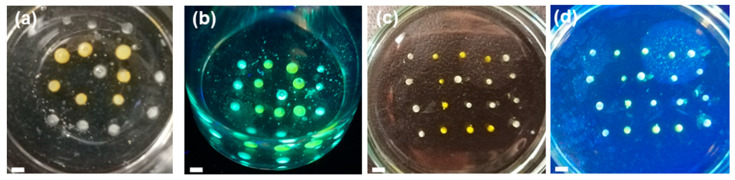
(**a**) Digital and (**b**) fluorescent images of colored pattern with CN/TCM and CN-F/TCM in aqueous PEI solution; (**c**) Digital and (**d**) fluorescent images of colored ‘c’ pattern with CN-A/TCM and CN-F/TCM in aqueous PEI solution. Scale bars correspond to 1 cm.

One can use this approach to fabricate emission-based encoding systems (Figure 5). Multicolor emissive soft droplets can be fabricated with great ease, which entails scale-up approaches, and many potential applications lie beyond this strategy.

## 4. Discussion

The emission property of organic and macromolecular structures has been of great interest for bioimaging and displays. Many reports shed a light on the mechanism of emission, and easy adjustment of the emission color is a challenge to be addressed. Recently, materials such as perovskite can grant an access for tunable emission color based on exchanging halide ion in the structure, however the toxicity of lead is a great issue to be considered. For g-CN, in aqueous media the tunability of emission was demonstrated via utilization of tailor-made polymers. Emission is generally considered in homogeneous systems as well as colloidal dispersions, and macroscopic heterogeneous all-liquid systems are yet to be discovered.

Herein, we have utilized vTA-modified CMp, which has exceptional dispersibility in organic media. Due to its highly charged structure, careful injection of this dispersion into aqueous PEI solution results in shape-persistent soft materials due to interfacial polyelectrolyte formation. High intensity emission arises from CMp-vTA and interfacial film formation renders stability to injected structures over a wide pH range.

Furthermore, the addition of polycyclic aromatic molecules into CMp-vTA dispersion promises an altered emission color and intensity. One can form multicolor emission patterns as well as apply realistic printing conditions to access a variety of shapes. An all-liquid large-scale heterophase emission platform is reported by facile chemistry that is extremely easy to conduct. 

## 5. Conclusions

Stable organic dispersions of polymeric g-CN were injected in an aqueous polycation solution, and the droplet shape was retained under water due to interface complexation of the two polymer compounds. So-formed liquid patterns show high fluorescence arising from g-CN, and the color could be facilely tuned by the addition of commodity polycyclic aromatic molecules. Such all-liquid systems could be used as multicolor emissive encoding platforms.
